# *IKZF1^plus^* is a frequent biomarker of adverse prognosis in Mexican pediatric patients with B-acute lymphoblastic leukemia

**DOI:** 10.3389/fonc.2024.1337954

**Published:** 2024-04-03

**Authors:** Joaquin Garcia-Solorio, Juan Carlos Núñez-Enriquez, Marco Jiménez-Olivares, Janet Flores-Lujano, Fernanda Flores-Espino, Carolina Molina-Garay, Alejandra Cervera, Diana Casique-Aguirre, José Gabriel Peñaloza-Gonzalez, Ma. Del Rocío Baños-Lara, Ángel García-Soto, César Alejandro Galván-Díaz, Alberto Olaya-Vargas, Hilario Flores Aguilar, Minerva Mata-Rocha, Miguel Ángel Garrido-Hernández, Juan Carlos Solís-Poblano, Nuria Citlalli Luna-Silva, Lena Sarahi Cano-Cuapio, Pierre Mitchel Aristil-Chery, Fernando Herrera-Quezada, Karol Carrillo-Sanchez, Anallely Muñoz-Rivas, Luis Leonardo Flores-Lagunes, Elvia Cristina Mendoza-Caamal, Beatriz Eugenia Villegas-Torres, Vincent González-Osnaya, Elva Jiménez-Hernández, José Refugio Torres-Nava, Jorge Alfonso Martín-Trejo, María de Lourdes Gutiérrez-Rivera, Rosa Martha Espinosa-Elizondo, Laura Elizabeth Merino-Pasaye, María Luisa Pérez-Saldívar, Silvia Jiménez-Morales, Everardo Curiel-Quesada, Haydeé Rosas-Vargas, Juan Manuel Mejía-Arangure, Carmen Alaez-Verson

**Affiliations:** ^1^ Laboratorio de Diagnóstico Genómico, Instituto Nacional de Medicina Genómica (INMEGEN), Mexico City, Mexico; ^2^ Unidad de Investigación Médica en Epidemiología Clínica, Unidad Medica de Alta Especialidad (UMAE) Hospital de Pediatría, Centro Médico Nacional (CMN) Siglo XXI, Instituto Mexicano del Seguro Social (IMSS), Mexico City, Mexico; ^3^ Subdirección de Genómica Poblacional, Instituto Nacional de Medicina Genomica (INMEGEN), Mexico City, Mexico; ^4^ Laboratorio de Citómica del Cáncer Infantil, Centro de Investigación Biomédica de Oriente, Instituto Mexicano del Seguro Social, Delegación Puebla, Puebla, Mexico; ^5^ Consejo Nacional de Humanidades, Ciencias y Tecnologías (CONAHCYT), Mexico City, Mexico; ^6^ Servicio de Onco-Pediatría, Hospital Juárez de México, Secretaría de Salud (SSA), Mexico City, Mexico; ^7^ Centro de Investigación Oncológica Una Nueva Esperanza, Universidad Popular Autónoma del Estado de Puebla, Puebla, Mexico; ^8^ Hospital General Centro Médico La Raza, Instituto Mexicano del Seguro Social (IMSS), Mexico City, Mexico; ^9^ Departamento de Oncologia, Instituto Nacional de Pediatría (INP), Mexico City, Mexico; ^10^ Departamento de Inmunogenetica, Instituto de Diagnostico y Referencia Epidemiologicos (InDRE), Mexico City, Mexico; ^11^ Unidad de Investigación Médica en Genética Humana, Hospital de Pediatría, CMN Siglo XXI, Instituto Mexicano del Seguro Social (IMSS), Mexico City, Mexico; ^12^ Servicio de Oncohematología Pediátrica, Hospital para el Niño Poblano, Secretaría de Salud (SS), Puebla, Mexico; ^13^ Servicio de Oncohematología Pediátrica, Instituto Mexicano del Seguro (IMSS) Unidad Médica de Alta Especialidad (UMAE) Centro Médico Nacional (CMN) Hospital de Especialidades Dr. Manuel Ávila Camacho, Puebla, Mexico; ^14^ Servicio de Hemato-Oncología Pediátrica, Hospital de la Niñez Oaxaqueña "Dr. Guillermo Zárate Mijangos", Secretaria de Salud y Servicios de Salud Oaxaca (SSO), Oaxaca, Mexico; ^15^ Hospital Infantil de Tlaxcala, Servicio de Oncología Pediátrica, Tlaxcala, Mexico; ^16^ Instituto de Seguridad y Servicios Sociales de los Trabajadores al Servicio de los Poderes del Estado (ISSSTE) de Puebla, Departamento de Enseñanza e Investigació, Puebla, Mexico; ^17^ Servicio de Oncología, Hospital Pediátrico Moctezuma, Secretaría de Salud de la Ciudad de México (SSCDMX), Mexico City, Mexico; ^18^ Servicio de Hematología, Centro Médico Nacional Siglo XXI, Instituto Mexicano del Seguro Social (IMSS), Unidad Médica de Alta Especialidad (UMAE) Hospital de Pediatría “Dr. Silvestre Frenk Freund”, Mexico City, Mexico; ^19^ Servicio de Oncología, Centro Médico Nacional Siglo XXI, Instituto Mexicano del Seguro Social (IMSS), Unidad Médica de Alta Especialidad (UMAE) Hospital de Pediatría “Dr. Silvestre Frenk Freund”, Mexico City, Mexico; ^20^ Servicio de Hematología Pediátrica, Hospital General de México, Secretaría de Salud (SSA), Mexico City, Mexico; ^21^ Servicio de Hematología Pediátrica, Centro Médico Nacional (CMN) “20 de Noviembre”, Instituto de Seguridad Social al Servicio de los Trabajadores del Estado (ISSSTE), Mexico City, Mexico; ^22^ Laboratorio de Medicina de Precisión, Instituto Nacional de Medicina Genómica (INMEGEN), Mexico City, Mexico; ^23^ Departamento de Bioquímica, Escuela Nacional de Ciencias Biológicas, Instituto Politecnico Nacional (IPN), Mexico City, Mexico; ^24^ Laboratorio de Genómica Funcional del Cáncer, Instituto Nacional de Medicina Genómica (INMEGEN), Mexico City, Mexico; ^25^ Facultad de Medicina, Universidad Nacional Autónoma de México (UNAM), Mexico City, Mexico

**Keywords:** *IKZF1^plus^*, *IKZF1* gene mutation, *CDKN2A/2B* gene mutation, *PAX5* gene mutation, PAR1 deletions, pediatric B-ALL, overall survival

## Abstract

**Background:**

Recurrent genetic alterations contributing to leukemogenesis have been identified in pediatric B-cell Acute Lymphoblastic Leukemia (B-ALL), and some are useful for refining classification, prognosis, and treatment selection. *IKZF1^plus^* is a complex biomarker associated with a poor prognosis. It is characterized by *IKZF1* deletion coexisting with *PAX5*, *CDKN2A/2B*, or PAR1 region deletions. The mutational spectrum and clinical impact of these alterations have scarcely been explored in Mexican pediatric patients with B-ALL. Here, we report the frequency of the *IKZF1^plus^* profile and the mutational spectrum of *IKZF1, PAX5, CDKN2A/2B*, and *ERG* genes and evaluate their impact on overall survival (OS) in a group of patients with B-ALL.

**Methods:**

A total of 206 pediatric patients with *de novo* B-ALL were included. DNA was obtained from bone marrow samples at diagnosis before treatment initiation. A custom-designed next-generation sequencing panel was used for mutational analysis. Kaplan-Meier analysis was used for OS estimation.

**Results:**

We identified the *IKZF1^plus^* profile in 21.8% of patients, which was higher than that previously reported in other studies. A significantly older age (*p=0.04*), a trend toward high-risk stratification (*p=0.06*), and a decrease in 5-year Overall Survival (OS) (*p=0.009*) were observed, although heterogeneous treatment protocols in our cohort would have impacted OS. A mutation frequency higher than that reported was found for *IKZF1* (35.9%) and *CDKN2A/2B* (35.9%) but lower for *PAX5* (26.6%). *IKZF1^MUT^* group was older at diagnosis (*p=0.0002*), and most of them were classified as high-risk (73.8%, *p=0.02*), while patients with *CDKN2A/2B^MUT^* had a higher leukocyte count (*p=0.01*) and a tendency toward a higher percentage of blasts (98.6%, >50% blasts, *p=0.05*) than the non-mutated patients. A decrease in OS was found in *IKZF1^MUT^* and *CDKN2A/2B^MUT^* patients, but the significance was lost after *IKZF1^plus^* was removed.

**Discussion:**

Our findings demonstrated that Mexican patients with B-ALL have a higher prevalence of genetic markers associated with poor outcomes. Incorporating genomic methodologies into the diagnostic process, a significant unmet need in low- and mid-income countries, will allow a comprehensive identification of relevant alterations, improving disease classification, treatment selection, and the general outcome.

## 1 Introduction

Genomic and transcriptomic analyses performed on large cohorts of pediatric and adult B-ALL patients have identified recurrent genetic alterations and expression signatures that contribute to leukemogenesis. Some of these have clinical utility as biomarkers for refining disease classification and treatment selection. The last WHO classification for B-lymphoblastic leukemia/lymphoma comprises 13 subtypes, depending on the specific genetic alterations acquired in the leukemic cells (1). In addition to gene fusion information, specific treatment protocols currently incorporate a combined evaluation of copy number alterations (CNAs) in the selected genes. CNAs affecting one or more genes related to cell differentiation, cell cycle control, and apoptosis have been identified in approximately 71% of pediatric B-ALL cases (2). Some CNA combinations, such as the *IKZF1^plus^* profile, have been identified as adverse modifiers of childhood B-ALL prognosis (3).

*IKZF1^plus^* is a high-risk category identified by Stanulla M et al. (3) in a cohort of 991 patients with B-ALL enrolled in the International Multicenter Trial AIEOP-BFM ALL 2000. *IKZF1^plus^* was defined as “deletion of *IKZF1* that co-occurred with at least one additional deletion in *CDKN2A, CDKN2B* (homozygous deletion only), *PAX5*, or PAR1 in the absence of *ERG* deletion”. The PAR1 region (pseudoautosomal region 1) is located in Xp22 and Yp11 and comprises the *CRLF2*, *CSF2RA*, and *IL3RA* genes. Patients with deletions affecting *ERG* were excluded from the *IKZF1^plus^* group. *IKZF1^plus^* was associated with a very poor prognosis in B-ALL patients with detectable minimal residual disease (MRD) (3). Following the initial report, several other studies have confirmed that this subgroup of patients had inferior outcomes (4–7).

*IKZF1* encodes Ikaros, a zinc-finger transcription factor required for the development of all lymphoid lineages. Deletions (*IKZF1^DEL^*) and small mutations in this gene lead to the acquisition of a stem cell-like phenotype by haploinsufficiency and loss of DNA-binding capacity (8). These alterations have been reported to be independent biomarkers of adverse prognoses (9–11). Deletions in the *ERG* gene (*ERG^DEL^*) can suppress this negative outcome, whereas JAK-STAT activation enhances this adverse effect (12, 13). These findings support the importance of evaluating the mutational profiles of several genes rather than specific alterations in isolated genes for more accurate risk stratification.

*CDKN2A* and *CDKN2B* (*CDKN2A/2B*) are tumor suppressor genes located on chromosome 9p21 and encode three key cell cycle regulators, p16INK4A and p14ARF, encoded by the alternative readings of *CDKN2A*, and p15INK4B, encoded by *CDKN2B* (14). *CDKN2A/2B* deletions (*CDKN2A/2B^DEL^*) have been detected in approximately 20–25% of pediatric patients with B-ALL (15). It has been suggested that inactivation of *CDKN2A/2B* is a secondary cooperative event that plays an essential role in leukemogenesis, cell cycle regulation, chemosensitivity, and apoptosis (14). The contribution of this alteration to prognosis is controversial. Some studies support that *CDKN2A/2B^DEL^*, especially in the case of biallelic status, is associated with inferior outcomes in B-ALL (16–18), while others claim that this alteration is not a poor prognostic factor in childhood B-ALL (14, 19).

*PAX5* encodes a transcription factor that participates in the development of normal B cells and in maintaining cell identity by repressing the signature genes of other lineages during differentiation (20). *PAX5* is found in a physiological complex with *IKZF1* and *RUNX1* (21). Somatic or germline alterations that deregulate *PAX5* activity may lead to B cell malignancies (22). *PAX5* alterations include deletions (*PAX5^DEL^*), focal intragenic amplifications (*PAX5^igAMP^*), translocations with various partners, or point mutations. All these were identified in B-ALL at different frequencies (23). Alterations in *PAX5* have been associated with inferior outcomes (2), which are worse when *IKZF1* deletion is present (24).

The frequency of alterations, mutational spectrum, and clinical impact of *IKZF1, PAX5, CDKN2A/2B*, *ERG* gene mutations, and the *IKZF1^plus^* profile have not been extensively explored in Mexican pediatric B-ALL patients. A better understanding of genomic alterations occurring in B-ALL pediatric leukemia and their clinical impact is crucial for Hispanic populations such as Mexicans. High incidence rates of childhood acute leukemia have been consistently observed in the Mexican population (55.0 cases per million children under 15 years of age) (25–27). Additionally, mortality and morbidity rates are higher than those in high-income countries, particularly as a consequence of deaths related to refractory disease and treatment-related toxicity (28, 29). The identification of clinically relevant genetic alterations may contribute to more effective and personalized treatment options for these patients, potentially improving their outcomes while minimizing unnecessary treatment-related side effects and costs. Genomic alterations play a significant role in determining disease evolution and the response of cancer cells to specific treatments (30). Genetic data from ethnically diverse populations would help obtain a more comprehensive understanding of leukemia subtypes and treatment responses across diverse populations.

This study aimed to identify the frequency and heterogeneity of the *IKZF1^plus^* profile and mutational spectrum of *IKZF1, PAX5, CDKN2A/2B*, and *ERG* in a cohort of 206 Mexican pediatric patients with *de novo* B-ALL. The clinical impact on overall survival was also evaluated for the analyzed genes and *IKZF1^plus^* profile.

## 2 Materials and methods

### 2.1 Population

This analysis included 206 *de novo* B-ALL pediatric Mexican patients from 10 different states of Mexico (**Supplementary Table 1A** displays the number of patients by state). Patients were treated at 18 public health institutions (**Supplementary Table 1B**). The diagnosis was established between January 2018 and April 2023 by pediatric hematologists/oncologists according to clinical features: cell morphology, immunophenotype, and genetics, as defined by the 2008 WHO classification of lymphoid neoplasms (31). The patients had a range of 0 to 17 years, and only 3 of them were younger than one year. The risk classification was established as defined by the Children’s Oncology Group (COG) and the National Cancer Institute (NCI) (32). The presence of at least one of the following clinical features was considered as high-risk: age at diagnosis ≥10 years, WBC counts higher than 5x10^4^ count/µL, positive minimal residual disease (MRD) on day 28, presence of nervous system infiltration, treatment failure response, and identification of any high-risk gene fusion (when that information was available). Cytogenetic results were unavailable for most patients. Before treatment initiation, 3–5 mL of bone marrow was collected into EDTA tubes at their respective medical institutions and sent to the Laboratory of Genomic Diagnosis at INMEGEN. Clinical data were collected from medical charts, including the child’s sex, age, white blood cell count in the peripheral blood, and percentage of blasts in the bone marrow at diagnosis and treatment protocol. The median follow-up of these patients was 1.4 years.

### 2.2 Nucleic acid extraction and quantification

DNA was isolated using the Maxwell® RSC Instrument (Promega Corporation, Madison, WI, USA). DNA concentration and quality were evaluated using a NanoDrop® 2000 spectrophotometer (Thermo Fisher Scientific Inc., Waltham, MA, USA) and a Qubit® 4 Qubit 1X dsDNA HS Assay Kit (Thermo Fisher Scientific Inc., Waltham, MA, USA).

### 2.3 Next-generation sequencing (Targeted DNAseq) and bioinformatics

A customized panel was designed to identify genetic alterations in *CDKN2A*, *CDKN2B*, *PAX5*, *ERG*, and *IKZF1*. Panel synthesis was performed using an Archer Dx (ArcherDX, Inc., Boulder, CO, USA). Library preparation was performed following the manufacturer’s instructions. Libraries were sequenced on the NextSeq 500/550 using the High Output Sequencing Reagent Kit v2.5 (300 cycles) (Illumina, Inc., San Diego, CA, USA). Bioinformatics analysis was performed using the Archer Suite Analysis v5.1.3 software (ArcherDX, Inc., Boulder, CO, USA) using the human reference genome GRCh37.p13/hg19. The bioinformatics pipeline allowed the identification of single nucleotide variations, small insertions/deletions, copy number alterations (CNAs), and structural variations (SV) (this last only for the *IKZF1* gene). According to the Archer Dx bioinformatics pipeline (ArcherDX, Inc., Boulder, CO, USA), SV occurs when individual reads contain nucleotide sequences that are aligned to different genome regions. The length of the SV was determined by observing the breakpoint positions of the partners identified in the event and subtracting the differences in their genomic positions (User Manual Archer Analysis 6.0 CS001). Mutations were considered if the variant allele fraction (VAF) was ≥ 5%. A minimum 500x-fold depth coverage was required for all targeted regions in the panel.

### 2.4 Multiplex ligation-dependent probe amplification

NGS-predicted *IKZF1, CDKN2A/2B*, and *PAX5* deletions were confirmed by MLPA using SALSA Probemix P335 (ALL-IKZF1) according to the manufacturer’s protocol (Holland, Amsterdam, Netherlands). This probe also allowed for the identification of PAR1 deletions, as described previously (3). The P327 iAMP21-ERG probe mix was used to confirm ERG deletions. Fragment separation was performed on a 3500 Genetic Analyzer (Thermo Fisher Scientific Inc., Waltham, MA, USA). The raw data files were imported and analyzed using Coffalyser.Net™ software (MRC Holland, Amsterdam, Netherlands).

### 2.5 *IKZF1^plu^* ^s^ profile definition

The *IKZF1^plu^* ^s^ profile was defined based on the *Stanulla et al., 2018* report (3). Briefly, the patient was considered *IKZF1^plus^* positive if *IKZF1^DEL^* co-occurred with *CDKN2A^DEL^*, *CDKN2B^DEL^* (homozygous deletion only), *PAX5^DEL^*, or pseudoautosomal region 1 (PAR1) deletion. As it has been reported that *ERG^DEL^* mitigates the adverse prognosis associated with *IKZF1* deletions (12), patients with *ERG^DEL^* were excluded from the *IKZF1^plus^* group. The *NO-IKZF1^plus^* group included any other patient, regardless of whether they were *IKZF1^MUT^* or *IKZF1^WT^*.

### 2.6 Statistical analysis

Measures of central tendency were used to describe continuous variables related to the patient’s clinical and demographic features. The χ² test or Fisher’s exact test was used to assess the relationship between categorical variables. In contrast, the nonparametric Mann–Whitney U test was used to analyze the association between continuous variables, where p <0.05 was considered statistically significant. OS was estimated using the Kaplan-Meier method. The log-rank test was used to evaluate the differences between survival distributions with a 95% confidence interval (CI). The Cox regression model was used to perform a multivariate analysis, with adjustments made for other risk factors such as sex, age at diagnosis, WBC count, percentage of blast cells, and risk stratification at diagnosis. OS was calculated from the day of diagnosis until either the last follow-up or death from any cause. Patients who did not experience any event were censored at the last follow-up visit. Those who did not attend the follow-up were censored at the date of the last known contact. Statistical calculations were performed using the RStudio software version 4.3.1, and the data were visualized using the ggplot2 package version 3.4.3.

## 3 Results

The clinical features of the study population are summarized in **Table 1**. Most patients (63.2%) were classified as high-risk. The most frequent high-risk features were age at diagnosis < 1 or ≥ 10 years, WBC counts higher than 5x10^4^ count/µL, followed by positive minimal residual disease (MRD) on day 28. The remaining patients were classified as having a standard risk.

**Table 1:** Clinical features of the analyzed population.

Characteristic	N (%)
**Sex**	**N=206**
Male	110 (53.4%)
Female	96 (46.6%)
**Age at diagnosis** **Mean 7.9 ± 4.8 (0-17 years)**	**N=185**
< 10	114 (61.6%)
≥ 10	71 (38.4%)
**WBC at Diagnosis (count/µL)**	**N=185**
< 10,000	82 (44.3%)
10,000 to < 20,000	36 (19.5%)
20,000 < 100,000	53 (28.6%)
≥ 100000	14 (7.6%)
**% of blasts at diagnosis in bone marrow**	**N=185**
20% to 50%	14 (7.6%)
≥ 50%	171 (92.4%)
**Risk classification at diagnosis**	**N=182**
Standard	67 (36.8%)
High Risk	115 (63.2%)
**High-risk criteria**	N (%)*
Age WBC Count Positive MRD Extramedullary disease Steroid pretretment Positive BCR::ABL1 Hypodiploidy Immunophenotype Not Specified	58 (50.43%) 34 (29.6%) 13 (11.3%) 12 (10.4%) 8 (6.9%) 6 (5.2%) 4 (3.5%) 3 (2.6%) 4 (3.5%)

WBC, white blood cell count; N, patients with available data for each category.

*Percentage of patients who met each high-risk criterion was calculated considering N=115 classified as “high-risk”, several patients had more than one, therefore percentage added>100%.

This group of patients was treated using six different protocols, depending on the medical preferences and institutional resources available in each case. The number of patients per treatment protocol is presented in **Supplementary Table 2**.

### 3.1 Frequency of *IKZF1^plus^* profile in B-ALL Mexican pediatric patients

The *IKZF1^plus^* profile was present in 21.8% (45/206) of the evaluated patients. Gene deletion combinations within the *IKZF1^plus^* group are shown in **Table 2**. In our series, three patients were excluded from the *IKZF1^plus^* profile because of *ERG^DEL^*.

**Table 2:** Frequency of the different gene-deletion combinations conforming to the *IKZF ^plus^* profile.

	*CDKN2A/2B*	*PAX5*	*PAR1*	N (%)
***IKZF1^DEL^***	DEL	NEG	NEG	15 (33.3%)
	DEL	DEL	NEG	13 (28.9%)
	NEG	DEL	NEG	7 (15.6%)
	DEL	NEG	DEL	5 (11.1%)
	NEG	NEG	DEL	4 (8.9%)
	DEL	DEL	DEL	1 (2.2%)
	*Total with IKZF1^plus^*			*45/206, (21.8%)*

N, patients in each category.

### 3.2 Clinical impact and overall survival analysis of the *IKZF1^plus^* profile

The patients were stratified according to the presence of *IKZF1^plus,^* and the clinical feature distribution was evaluated between the groups (**Table 3**). Age at diagnosis showed significant differences between the groups, with patients with *IKZF1^plus^* being older (*p=0.04*). Additionally, a trend toward higher risk classification was observed within the *IKZF1^plus^* group. No significant differences were found in sex distribution, percentage of bone marrow blasts, or WBC count at diagnosis between the two groups.

**Table 3:** Clinical features of patients with the *IKZF1^plus^* profile.

Characteristics	*IKZF1^plus^*	*NO IKZF1^plus^*	OR CI 95%	*p value
**Sex**	**N=45**	**N=161**		
Male	28 (62.2%)	84 (52.2%)	1.5098 0.7669 to 2.9723	0.23
Female	17 (37.8%)	77 (47.8%)		
**Age at diagnosis**	**N=38**	**N=149**		
< 10	18 (47.4%)	97 (65.1%)	2.0726 1.0085 to 0.42598	0.04
≥ 10	20 (52.6%)	52 (34.9%)		
**WBC at diagnosis**	**N=38**	**N=147**		
< 10,000	15 (39.5%)	67 (45.6%)		
10,000 to < 20,000	6 (15.7%)	29 (19.7%)	0.9241 0.3259 to 2.6203	0.82
20,000 < 100,000	14 (36.8%)	39 (26.5%)	1.6034 0.7002 to 3.6715	0.26
≥ 100000	3 (8%)	12 (8.2%)	1.1167 0.2799 to 4.4543	0.87
**% of blasts at diagnosis in bone marrow**	**N=38**	**N=147**		
20% to 50%	0 (0%)	13 (8.8%)	7.7286 0.4491 to 132.9930	0.15
≥ 50%	38 (100%)	134 (91.2%)		
**Risk classification at Diagnosis**	**N=38**	**N=144**		
Standard	9 (23.7%)	58 (40.3%)	2.1731 0.9584 to 4.9275	0.06
High Risk	29 (76.3%)	86 (59.7%)		

*Fisher’s exact or chi-square test; OR, Odds Ratio; CI, Confidence interval; N, number of patients with available data for each category; WBC, white blood cell count.

**Figure 1:**
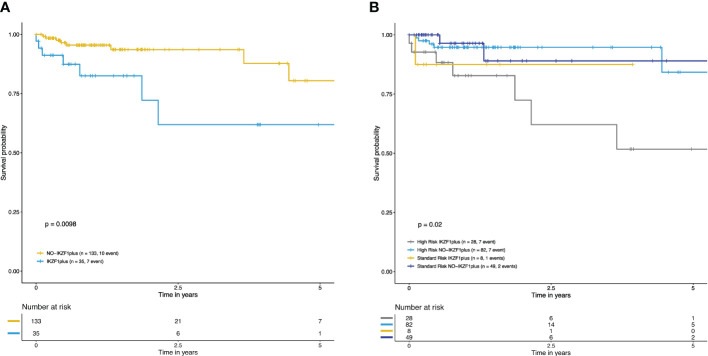
Overall survival curves at five years of *IKZF1^plus^* were estimated with the Kaplan–Meier method. **(A)** Decreased OS in patients with *IKZF1^plus^* (blue) versus *NO-IKZF1^plus^* (yellow). **(B)** Overall survival curves stratified according to *IKZF1^plus^* status and risk classification at diagnosis. The lowest OS was observed in the high-risk *IKZF1^plus^* positive group. Standard-risk *NO-IKZF1^plus^* patients (navy blue), high-risk *NO-IKZF1^plus^* patients (light blue), high-risk *IKZF1^plus^* (gray), standard-risk *IKZF1^plus^* (yellow).

The impact of *IKZF1^plus^* on OS at five years was evaluated in 168 patients for whom information for OS calculation was available (35 *IKZF1^plus^* vs. 133 *NO-IKZF1^plus^*). The *IKZF1^plus^* group showed significantly reduced OS at 5 years compared to the *NO-IKZF1^plus^* group (*p=0.009*, **Figure 1A**). A significantly worse OS was also observed for the high-risk-*IKZF1^plus^*-positive subgroup when patients were stratified by the *IKZF1^plus^* status and the risk category (*p=0.02*, **Figure 1B**).

We conducted a Cox regression multivariate analysis to examine the impact of the *IKZF1^plus^* profile, taking into account additional clinical factors such as patient age, sex, white blood cell count, percentage of blast cells, and the risk classification at the time of diagnosis. The analysis revealed that having the *IKZF1^plus^* profile significantly increases the risk of death by 3.7 times (HR = 3.7227, p = 0.02), highlighting its role as an independent poor outcome-predicting factor. The other variables included in the model did not demonstrate a statistically significant effect on the outcome.

### 3.3 Mutational profiles of *IKZF1*, *PAX5*, *CDKN2A*, *CDKN2B*, *ERG* genes and PAR1 regions in Mexican patients with B-ALL

**Figure 2:**
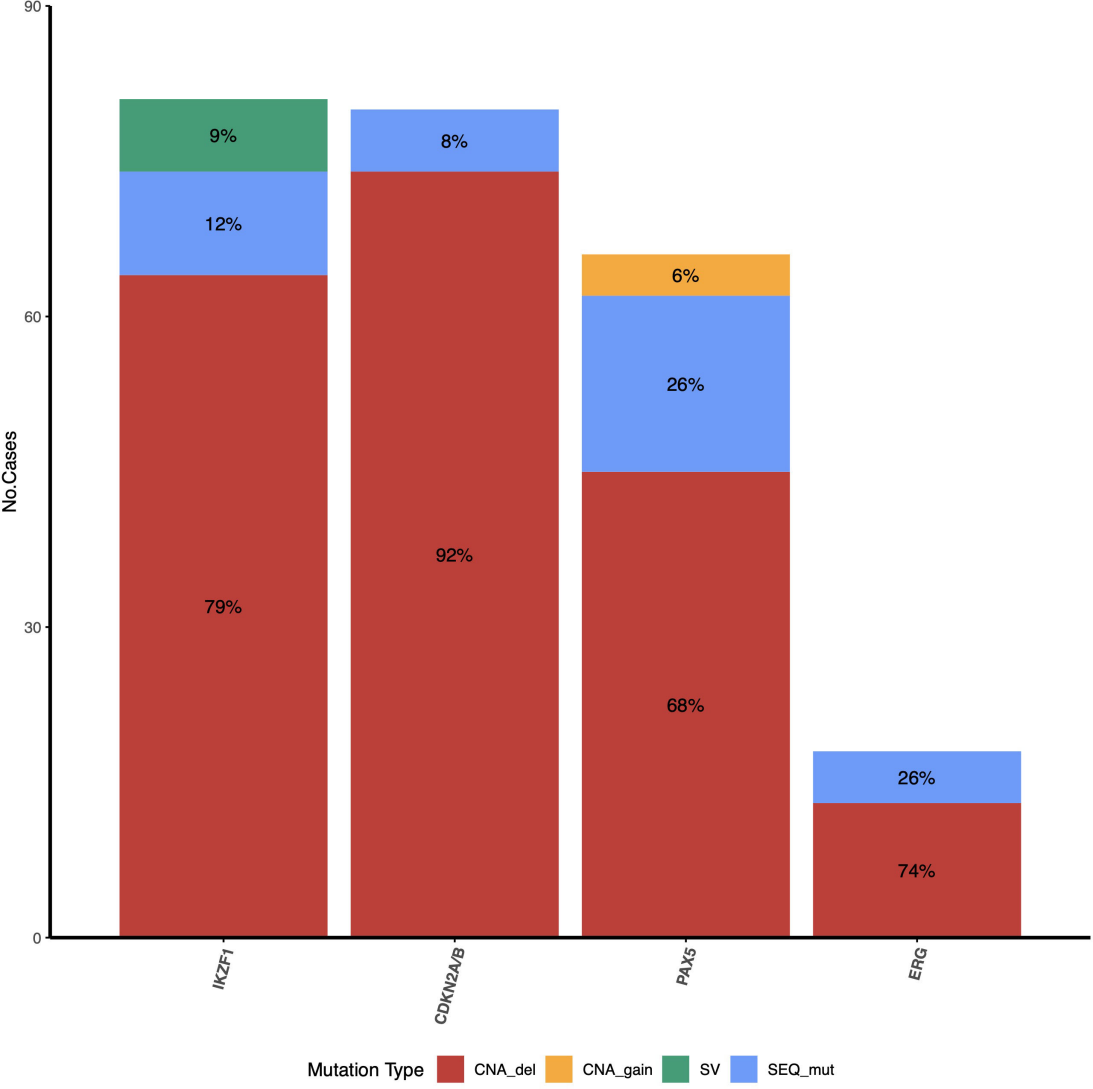
Frequency and type of mutations identified for *IKZF1*, *CDKN2A/2B*, *PAX5*, and *ERG* genes. For *IKZF1*, CNAs concomitant (red) with SV (green) were identified in some patients (**Supplementary Table 4**); these patients were counted only in the CNA_del group in this figure. The PAR1 region was not included because only deletions can be detected by MLPA.

**Figure 3:**
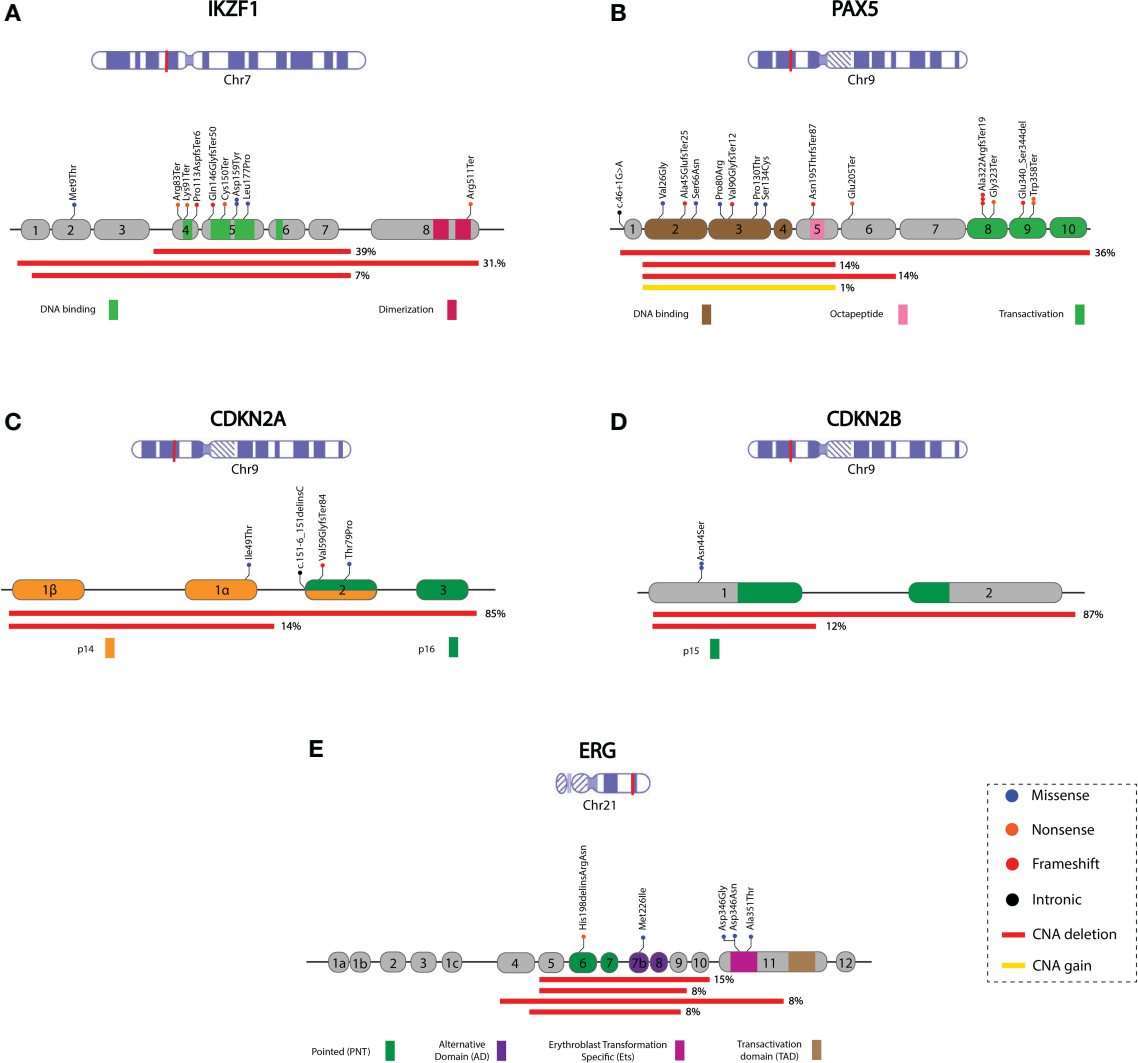
Schematic representation of the mutational profile identified in the *IKZF*1, *PAX5*, *CDKN2A*, *CDKN2B*, and *ERG* genes. Coding exons and the corresponding protein domain are shown for each gene. Red lines indicate the deleted region, and the percentage (right side) represents the proportion of cases with deletions. The remaining percentage corresponds to deleted regions with lower frequencies (not represented in the Figure). The complete deletion information is available in **Supplementary Table 4**. **(A)** *IKZF1*; NM_006060, **(B)** *PAX5*; NM_016734.2, the yellow line represents regions with *PAX5^igAMP^*, **(C)** *CDKN2A*; NM_000077, **(D)** *CDKN2B*; NM_004936, **(E)** *ERG*; NM_004449. Chromosome figures were taken from NCBI Bookshelf ID: NBK22266.

The types of mutations identified in each gene and their frequencies are shown in **Figure 2**. *IKZF1* was altered (*IKZF1^MUT^*) in 35.9% (74/206) of patients studied (**Supplementary Table 3** for sequence mutations and **Supplementary Table 4** for CNAs). Deletions were the most common alterations, representing 86.5% (64/74) of positive cases. Deletion affecting exons 4–7 (IK6 isoform) was the most common (39%, 25/64), followed by complete gene deletion (31.2%, 20/64). Moreover, a deletion spanning exons 1–8 was present in 7.9% (5/64) of cases (**Figure 3A**). SV was also detected in 54.7% of the patients with deletions. In contrast, only SVs were identified in seven individuals; interestingly, in six of them, the VAF was < 10%.

Sequence mutations were identified in 9 cases; in six of them, a concomitant deletion or SV was also present. Ten mutations were identified in nine cases: four missense mutations, four nonsense mutations, and two frameshift mutations, most of which were located in the DNA-binding domain coding region. In two patients, p.Asn159Tyr (N159Y) was identified without additional *IKZF1* alterations.

At least one *PAX5* alteration (*PAX5^MUT^*) was identified in 26.6% (55/206) of patients (**Supplementary Tables 3, 4**). These include CNAs caused by complete or partial gene deletions, gains, and small sequence mutations. *PAX5^DEL^* was the most common alteration, representing 75% (41/55) of *PAX5^MUT^*. In most cases, 63.4% (26/41), several exons were deleted, most commonly from exon 2 to 5 or from exon 2 to 6 (14%, 6/41 of each). In two cases, only one exon was deleted (exon 1 or exon 7). The whole gene (exons 1-10) was deleted in 36.6% (15/41) of cases. *PAX5^igAMP^* was identified in four patients; in two of them, additional genetic material was present in exons 2-5, one in exon 5, and one in the whole gene (**Figure 3B**; **Supplementary Table 4**).

A total of 17 *PAX5* sequence mutations were identified in 15 patients: four nonsense mutations, five missense mutations [one p.Pro80Arg (P80R)], seven frameshifts, and one intronic variant (**Supplementary Table 3**). In eight of them, a second alteration in *PAX5* was also present: four deletions, two sequence mutations, and two gains of material in *PAX5*.

*CDKN2A^DEL^* was identified in 35.92% (74/206) of the cases; in most cases (65/74), *CDKN2B* was entirely or partially deleted (**Figures 3C, D**). *CDKN2A* homozygous deletion was observed in 40 patients; in 30 of them, *CDKN2B* also showed homozygous deletion. The most frequent *CDKN2A^DEL^* comprised exons 1 to 3 and was identified in 85.1% (63/74) of the patients, followed by the deletion of exon 1, which was identified in 14.9% (11/74) of *CDKN2A^DEL^* patients. For *CDKN2B*, exon 1 and 2 deletions were identified in 87.7% (57/65), followed by exon 2 deletion in 12.3% (8/65) of the *CDKN2B-*positive cases (**Supplementary Table 4**). In six cases, sequence variants were identified in *CDKN2A* (four cases) and *CDKN2B* (two cases) (**Supplementary Table 3**).

*ERG^MUT^* was identified in 8.7% (18/206) of cases (**Supplementary Tables 3, 4**). Among the *ERG^DEL^* patients (13/18), involved various exons. These deletions affect the region expanding exons 2 to 11, encoding the *ERG* protein’s pointed domain (PNT) or alternative domain (AD) of the ERG protein. Sequencing variants were identified in five cases, three of which were located in the erythroblast transformation-specific domain (Ets) of the protein (**Figure 3E**, **Supplementary Table 3**).

Deletion of the PAR1 region was assessed by MLPA in patients with *IKZF1^DEL^*. We identified only ten patients with PAR1 deletions (**Supplementary Table 4**).

### 3.4 Clinical impact and overall survival analysis of patients with *IKZF1*, *PAX5*, *CDKN2A/2B*, and *ERG* mutations

Sex distribution, age at diagnosis, WBC count at diagnosis, percentage of blasts, and risk assignment were compared between patients positive and negative for *IKZF1*, *PAX5*, *CDKN2A/2B*, and *ERG* mutations (**Supplementary Tables 5A–D**). The frequency of high-risk patients and of those older than 10 years at diagnosis was significantly higher in the *IKZF1^MUT^* group (*p=0.02* and *p=0.002*, respectively). A higher proportion (37.7%) of patients with *CDKN2A/2B^MUT^* had a WBC count in the range of 20,000–100,000 cells than *CDKN2A/2B^NEG^* patients (22.4%) (*p=0.01*). Additionally, a significantly higher proportion of patients had > 50% blasts at diagnosis in the CDKN*2A/2B^MUT^* (98.6%) group than in the *CDKN2A/2B^NEG^* group (89.7%) (*p=0.05*) (**Supplementary Table 5B**). In the case of *PAX5^MUT^*, a higher percentage of cases was associated with high-risk classification at diagnosis, but the difference was not statistically significant (*p=0.06*).

A significant decrease in OS was observed in the *IKZF1^MUT^* (*p=0.02*) (**Supplementary Figure 1A**) and *CDKN2A/2B^MUT^* (*p=0.006*) (**Supplementary Figure 1B**) groups. Significant differences (*p=0.02*) in OS were identified between the homozygous *CDKN2A^MUT^* and heterozygous *CDKN2A^MUT^* vs. *CDKN2A^NEG^* patients. (**Supplementary Figure 1C**). No significant effect on OS was observed for *PAX5^MUT^* or *ERG^MUT^* (**Supplementary Figures 1D, E**).

To determine whether the observed negative impact of *CDKN2A/2B^MUT^* and *IKZF1^MUT^* mutations on OS was conferred by *IKZF1^plus^*-positive patients in both groups, we excluded *IKZF1^plus^* patients from the OS analysis. After eliminating these patients, no significant differences in OS were observed between the *CDKN2A/2B^MUT^* or *IKZF1^MUT^* groups and those without mutations in these genes (**Supplementary Figures 2A, B**).

## 4 Discussion

To the best of our knowledge, this is the first study to evaluate the frequency, heterogeneity, and clinical impact of the *IKZF1^plus^* profile in an unselected group of Mexican pediatric patients with B-ALL. We also report the comprehensive mutational spectrum of *IKZF1*, *PAX5*, *CDKN2A/2B*, and *ERG* genes present at diagnosis in these patients. In contrast to most published studies that used MLPA to evaluate deletions in *IKZF1*, *PAX5*, *CDKN2A/2B*, and *ERG* genes related to the *IKZF1^plus^* profile, we used a custom-designed panel based on NGS. The bioinformatics algorithm allows the simultaneous evaluation of CNA (gain or deletions) with higher sensitivity than MLPA and of the sequence mutations present in at least 5% of the cells.

The frequency of *IKZF1^plus^* was higher (21.8%) than that reported in previous studies that analyzed patients of European ethnicities. Examples include Stanulla et al. (6%) (3), Braun et al. (2%) (4), Kicinski et al. (6%) (33), and Schwab et al. (13%) (34). Although few studies have been performed in Latin American populations, the frequencies are also lower than those detected in Argentina (9.2%) (5) and Brazil (11%, including pediatric and adult patients) (35). Using NGS, a more sensitive analysis methodology may have contributed to the higher frequency observed in our study. MLPA can only detect alterations if present in at least 25% of cells but fails to detect alterations when present in a smaller fraction of malignant cells, such as a subclone.

The two most frequent combinations, representing approximately 70% of the *IKZF1^plus^*-positive cases, comprised the simultaneous deletion of *IKZF1*-*CDKN2A/2B* or *IKZF1*-*CDKN2A/2B*-*PAX5*, similar to the results observed in other series (4). The high frequency of these two combinations is unsurprising given that *CDKN2A/2B^DEL^* is the most recurrently reported CNA in B-ALL, and frequently, the deletion also removes *PAX5*, which is located close to the *CDKN2A/2B* complex.

Because gene fusion information and iAMP21 were available for these patients (personal communication, the fusion data are being prepared for publication), we correlated the distribution of *IKZF1^plus^* with these alterations. The *BCR::ABL1* fusion group had the highest proportion of *IKZF1^plus^*-positive patients (66.7%), followed by the iAMP21 (50%) and Ph-like groups (47%). *IKZF1^plus^* was also present in cases without these alterations but in a much lower proportion (13.7%)*. IKZF1^plus^* was not identified in the *ETV6::RUNX1, TCF3::PBX1, PAX5::ETV6, DUX4, or MEFD2* rearranged group of patients. The *IKZF1^plus^* molecular profile was associated with older age at diagnosis (52.6% ≥ 10 years in the *IKZF1^plus^* group vs. 34.9% in the *NO-IKZF1^plus^* cases, *p=0.04*) and high-risk classification at diagnosis (76.3% of *IKZF1^plus^* were classified as high-risk, *p=0.06*), similar to previous findings (3, 36).

According to our results, *IKZF1^plus^* had a significantly negative effect on OS. Similar results were reported by Crepinsek et al., who showed that the *IKZF1^plus^* subgroup had the lowest OS compared with *IKFZ1^MUT^* patients or *IKZF1^WT^* (36). The decline in OS observed in our patients was especially pronounced for *IKZF1^plus^* patients classified as high-risk at diagnosis compared with *IKZF1^plus^* standard-risk patients. In most studies, the negative prognostic impact of *IKZF1^plus^* on disease-free survival (DFS) was found to be limited to the subgroup of patients with positive MRD after induction (4, 33). The effect of *IKZF1^plus^* on DFS was not evaluated in our patients because the MRD information was not available. However, a more recent study that included 1,200 patients reported no statistically significant association between *IKZF1^plus^* and DFS (33).

### 4.1 Mutational profile of *IKZF1*, *PAX5*, *CDKN2A*, *CDKN2B*, *ERG* genes and PAR1 region in Mexican patients with B-ALL

The frequency of *IKZF1^MUT^* in this cohort (35.9%) was higher than that reported for Mexican patients. Ayón-Perez et al. found that 20.6% (36 cases) (37), while Rosales-Rodríguez et al. (38) detected 27% (63 cases) both using MLPA. The frequency of *IKZF1^MUT^* in our study was still higher (31.1%) than previously reported in Mexicans if only the deletions were considered in the *IKZF1^MUT^* count. *IKZF1^MUT^* frequencies in other populations were also lower: American (28.6%) (9), Brazilian (19.3%) (39), and Argentinian (9.2%) (5). The differences in the sensitivity of the methodology contributed to the increased frequency detected in our study. In most previous reports, MLPA was used; therefore, SV present in a low proportion could not be observed. In fact, seven patients in our study had SV with a VAF < 10%; concomitant deletion was not detected in these patients. Therefore, they would have been considered “negative” if MLPA had been used as the detection method.

In most patients with *IKZF1^DEL^*, deletion of exons 4–7, which encode the dominant negative IK6 isoform, was identified. Whole gene deletion, resulting in the loss of expression of the wild-type allele, was the second most frequent deletion. Four patients showed deletions in the noncoding exon 1. Deletions involving the 5’ region of *IKZF1* also result in haploinsufficiency owing to a significant reduction in *IKZF1* mRNA expression (40). *IKZF1* is intolerant to variants that cause loss of function, according to the constraint metrics obtained from the gnomAD exome database (41).

The *IKZF1* ^N159Y^ mutation was present in less than 1% of our cases (two cases, 0.9%), and a similar low frequency was found in an analysis of 1988 pediatric B-ALL cases (0.4%) (42). No other *IKZF1* alterations were present in these two patients, suggesting retention of the non-mutated *IKZF1* allele, as previously reported in this subgroup of patients (42). This mutation is located inside the DNA-binding domain and induces nuclear mislocalization and intercellular adhesion. The transcriptomic profile of the *IKZF1* ^N159Y^ positives was different from that observed in other patients with alterations in *IKZF1*, suggesting that *IKZF1* ^N159Y^ could define a new subtype of B-ALL (42). The upregulation of genes involved in oncogenesis, chromatin remodeling, and signaling has been identified in patients with *IKZF1* ^N159Y^ (43).

*IKZF1^DEL^* was associated with older age at diagnosis, which is in accordance with the results of previous studies (9, 44). *IKZF1^DEL^* was associated with poor outcomes in several studies (9–11). In our patients, a significant decrease in OS was also observed for *IKZF1^MUT^* (*p=0.02*). Still, it seems to have been mainly contributed by the *IKZF1^plus^* patients present in the *IKZF1^MUT^* group since significance was lost after removing the *IKZF1^plus^* patients. In previous reports, the contribution of *IKZF1^plus^* to the poor outcome observed with *IKZF1^MUT^* was not independently evaluated.

*PAX5^MUT^* was less frequent in Mexican patients than in other ethnic groups. *PAX5^MUT^* has been found in approximately one-third of non-Hispanic B-ALL pediatric cases, according to studies performed in the USA and Netherlands (9, 45). In this study, the proportion of *PAX5^MUT^* was only 26.6%, considering all alterations (deletions, gain, and small sequence mutations). The frequency of *PAX5* fusion-positive patients increased to only 27.7%, which is still lower than that reported in other studies (46). A previous study in Mexican patients found that 15.9% *PAX5^DEL^* by MLPA (38) was lower than that reported in Brazilians (25.2%) using the same methodology (39).

Monoallelic deletions affecting the paired domain were the most frequently identified *PAX5* alterations, similar to the findings in other groups (9, 45). They involve small portions of the gene or cause whole-gene loss, impairing the DNA-binding capacity of the PAX5 protein. Mouse models have shown that haploinsufficiency of Pax5, caused by monoallelic deletion, confers susceptibility to B-cell transformation. However, other oncogenic events that act synergistically are required for leukemic transformation (47). In our cohort, *PAX5^DEL^* was concomitant with the BCR::ABL1, Ph-like-related fusions, and *EBF1* mutations. The activation of STAT5 and mutations in *EBF1, JAK3*, and *BCR::ABL1* have also been identified as cooperative events in *PAX5^DEL^* mice (48, 49).

Extra copies of *PAX5* regions were identified in four cases, representing 1.9% of the total and 7.4% of the *PAX5*-positive group. An extra chromosome 9 would be present in one of them, as the other genes in chromosome 9 included in the NGS panel also showed more than two copies. No cytogenetic data were available for this patient to confirm the presence of extra Chr 9. *PAX5^igAMP^* was the most likely alteration in the three other patients, with a gain in genetic material observed for the exons encoding the DNA-binding and octapeptide domains, as reported previously (50).

*PAX5^igAMP^* has been associated with male sex, age at diagnosis older than ten years, and high-risk stratification. This alteration is frequently associated with *CDKN2A/2B^DEL^* (82%), and appears to be mutually exclusive with other significant risk-stratifying genetic lesions (50). Despite the small number of patients with *PAX5^igAMP^* in our analysis (3 cases), the clinical features were similar to those reported: three were males, 2/3 were older than ten years, and 1/3 had nine years; all were classified as high-risk at diagnosis. *CDKN2A/2B^DEL^* was present in all cases, and *BCR::ABL1* was the only fusion identified (one case). Although the functional consequences of amplification have not yet been established, it has been suggested that increased copies of the DNA-binding region may alter binding to PAX5 target genes, leading to dysregulated B-cell differentiation and transformation (51). *PAX5^igAMP^* was associated with a high incidence of relapse (50). We did not evaluate the impact of *PAX5^igAMP^* on OS because of the small number of patients.

It has been suggested that some sequencing mutations may serve as initiating rather than secondary cooperative events in leukemogenesis (42). *PAX5* point mutations have been identified in 7–10% of pediatric cases of B-ALL, similar to the frequency in our patients (7.3%), being the second most common *PAX5* type of alteration. Fourteen mutations were identified, mainly located in the paired or transactivating domains. The most common consequence was the loss of function due to a frameshift or premature stop codon (7/14). Only seven of them have been previously reported in the COSMIC database (as of October 2023), and two previously described cases were recurrent in our patients: p.Ala322ArgfsTer19 (three cases) and p.Trp358Ter (two cases), both of which affect the transactivation domain. The frequency of *PAX5^P80R^* in our cases was lower than that previously reported. Only one case (0.5%) was identified, in contrast to the 2–6% observed in other studies (42, 52–54). In accordance with previous observations, this case was negative for fusions or iAMP21 and had an additional *PAX5* loss-of-function mutation affecting the other allele. Martínez-Anaya et al. reported *PAX5^P80R^* in 1.4% of the patients; however, only Ph-like Mexican pediatric patients were analyzed (55). In seven cases with sequence mutations, both *PAX5* copies were affected, with a deletion, gain, or a second point mutation in the other allele.

*PAX5* alterations can function as cooperative events or germline-initiating genetic lesions in B-ALL patients (23). In most of our cases, the VAF of *PAX5* sequence mutations was lower than expected, considering the percentage of blast cells present in the bone marrow sample analyzed, suggesting that *PAX5* sequence mutations are secondary events that occur only in a subpopulation of malignant cells. Additionally, none of the sequencing mutations identified in our study has been reported as germline variants according to the HGMD professional database (as of October 2023) or had a VAF suggesting a germline origin.

As observed in another series of B-ALL patients, *CDKN2A/2B^DEL^* was the most frequently identified CNA (35.9%) in Mexican patients. Homozygous deletion of both genes occurred in a high proportion of the cases (40.5%). As previously reported, none of the patients had *CDKN2B^DEL^* without *CDKN2A^DEL^* (3). The frequency of *CDKN2A/2B^DEL^* was higher than that reported by Rosales-Rodríguez et al. (31.7%) in Mexican patients (38). Lower frequencies have also been reported in other Latin American and Asian populations, including Colombia (25%) (56), Brazil (31.8%) (39), China (20.2%) (15), and India (19.8%) (57). In our study, *CDKN2A/2B^DEL^* was associated with higher blast counts at diagnosis and high-risk features. Most patients with this alteration were classified as high-risk, although the difference was not statistically significant.

Sequence variants of *CDKN2A/2B*, including *CDKN2A* (1.9%) and *CDKN2B* (0.97%), occur in a very low proportion of patients with B-ALL. Three *CDKN2A* sequence variants, p.Ile49Thr, p.Val51ProfsTer89, and c.151-6_151delinsC, had a VAF close to 50% and were classified as likely pathogenic according to ACMG criteria (58). However, p.Ile49Thr is probably of germline origin since it has been previously identified in the Latino population database (f=0.00451, 1456/34586, gnomAD exomes database, as of October 2023). Although it is listed in ClinVar (ID 127523) with conflicting classifications (pathogenic vs. uncertain significance), functional studies support its role in pathogenicity (59). However, this effect appears more moderate than other known pathogenic variants of *CDKN2A* (60). The variant has been reported to be pathogenic in families with melanoma and pancreatic cancer (60). It has been identified several times in Mexican women with breast cancer who fulfill genetic risk criteria. Ten patients with cancer carrying this variant, identified in our laboratory, reported no family history of leukemia or other hematological malignancies during routine evaluation for cancer predisposition syndromes. No history of cancer was present in the family of the B-ALL patient carriers of this variant. The variant p.Val51ProfsTer89 produces a loss of function due to a frameshift, and c.151-6_151delinsC is a deletion insertion that affects the canonical splicing site in exon two and may cause nonsense-mediated decay. These variants are not listed in the HGMD or ClinVar databases (as of October 2023), are absent from population databases, and have not been reported as germline variants in cancer syndromes. None of the patients had a family history of cancer. As DNA obtained from non-hematopoietic tissue was unavailable, variants’ germline or somatic origins could not be clarified.

*CDKN2B* p.Asn44Ser was identified in two cases, with a VAF of approximately 50%. It is classified as having uncertain significance according to ACMG and has not been reported as a somatic mutation. It has a low frequency in Latino individuals. (f=0.0121%, 4/33098, gnomAD exomes, as of August 2023), and was absent from the Mexican database incorporated into the Franklin By Genoox platform (470 individuals). The allele frequency in our cohort of B-ALL patients was significantly higher than in gnomAD patients (0.4% in B-ALL vs. 0.0121% in gnomAD exomes, X^2^ = 22, *p=2.7E-06*). As both patients were from Oaxaca State, we can speculate that this variant could be frequent in this region of Mexico. However, a possible association with an increased risk of leukemia development cannot be ruled out without further investigation.

OS was significantly decreased (*p=0.006*) in the *CDKN2A/2B^MUT^* group, but similar to what happened for the decreased OS in the *IKZF1^DEL^* group, the significance was lost after removing the *IKZF1^plus^* patients. *CDKN2A/2B^DEL^* has been correlated with inferior 3-year event-free survival and 3-year OS rates in B-ALL patients (15). Other researchers have concluded that *CDKN2A/2B^DEL^* is associated with an increased probability of relapse and death (17, 57, 61, 62), but the contribution of *IKZF1^plus^* patients to these outcomes has not been evaluated.

No conclusive data exist regarding the contribution of *CDKN2A/2A^DEL^* heterozygous or homozygous status. In our study, *CDKN2A/2B^DEL^* heterozygous patients showed a significant decrease in OS (*p=0.02*) compared with homozygous and wild-type patients. However, Feng et al. (15) showed that patients with biallelic deletions had a worse 3-year EFS than those without. However, other authors have reported that homozygous *CDKN2A/2B^DEL^* is not associated with poor prognosis in childhood B-ALL (63).

In our cohort, somatic mutations in *ERG* were identified in 8.7% of patients, with most presenting deletions (72.2% of the *ERG^MUT^* cases) that affect one or more exons encoding the *ERG* protein’s pointed domain of the ERG protein or the alternative domain. All sequence variants identified in *ERG* are probably somatic, considering the VAF, and are located mainly in the erythroblast transformation-specific domain. According to the COSMIC database (as of October 2023), these domains are reported to be mutated in hematopoietic neoplasms.

Patients with *ERG^MUT^* had better OS, although statistical significance was not reached because of the small sample size. None of the patients with *ERG^DEL^* experienced mortality, supporting the reported association between *ERG^DEL^* and favorable outcomes (12, 13).

The present study has several limitations that must be considered when interpreting the results. It was a multicenter retrospective analysis of a heterogeneous group of patients. Treatment protocol and hospital-related factors would have influenced OS. The patients were treated according to six protocols, the only target therapy available was imatinib, and it was used only in the BCR::ABL1 positive patients. Due to the lack of molecular testing, Ph-like patients were not identified and did not receive target therapy, which would affect their OS. Economic resources and hospital infrastructure may vary among the 18 hospitals where the patients were treated. Cytogenetic/FISH characterization and MRD determination are not routinely performed in all Mexican public institutions, and this information was unavailable for these patients. Despite being one of the most extensive series evaluating molecular alterations in pediatric B-ALL, the number of patients is still small, and clinical information was not available for 100% of the patients; thus, it reduces the statistical power to detect additional differences in the distribution of clinical features between mutation-positive and mutation-negative patients or between subgroups of *IKZF1^plus^* patients or to evaluate the impact of specific mutations on OS. Finally, additional biological and socioeconomic factors that were not assessed in the present study could have contributed to the poor patient outcomes.

## 5 Conclusion

This study is the first to evaluate the prevalence and impact of the *IKZF1^plus^* profile on OS in Mexican pediatric patients with B-ALL using NGS. Our results showed a higher frequency than previously reported and a significantly lower OS in patients with this alteration. Our results support the notion that a high frequency of genetic markers related to poor outcomes is present in Mexican patients at the time of diagnosis. The interplay between adverse genetic features and socioeconomic challenges contributes to the higher mortality and morbidity observed in pediatric B-ALL patients in our country (64, 65). The incorporation of genomic methodologies into the diagnosis process of B-ALL, a significant unmet need in low- and mid-income countries, will allow the comprehensive identification of clinically relevant alterations, improving disease classification, treatment selection, and probably the general outcome.

## Data availability statement

The data presented in this study are deposited in online repository of National Center for Biotechnology Information, ClinVar, accession number SUB13858637.

## Ethics statement

The studies involving humans were approved by Comisión Nacional de Investigación Científica del Instituto Mexicano del Seguro Social (IMSS) (Number of document R-2020-785-022. The studies were conducted in accordance with the local legislation and institutional requirements. Written informed consent for participation in this study was provided by the participants’ legal guardians/next of kin.

## Author contributions

JGS, JCNE, JMMA, and CAV carried out the conceptualization, data curation, formal analysis, funding acquisition, investigation, methodology, project administration, resources, software, supervision, validation, and visualization. MJO, FFE, CMG, AC, KCS, AMR, LLFL, ECMC, BEVT, VGO, ECQ, HFA, and JMMA provided technical support in methodology. JCNE, JFL, DCA, JGPG, EJH, MRBL, JRTN, AGS, JAMT, CAGD, MLGR, AOV, RMEE, LEMP, MLPS, MMR, MAGH, JCSP, NCLS, LSCC, PMAC, HRV, SJM, and FHQ provided resources such as study materials and clinical data. JGS and CAV undertook writing – original draft and writing – review & editing. Final approval of the manuscript was provided by all authors, who were accountable for all aspects of the work. JGS and JCNE share equal contributions, whereas JMMA and CAV are corresponding authors.
