# Supplementary material for: IKZF1plus is a frequent biomarker of adverse prognosis in Mexican pediatric patients with B-acute lymphoblastic leukemia
Source: Front Oncol. 2024 Apr 3;14:1337954. doi: 10.3389/fonc.2024.1337954 (PMC11022689; doi:10.3389/fonc.2024.1337954)
Supplement: Supplementary file 2 [file DataSheet_2.docx]

Supplementary Material

Joaquin Garcia-Solorio *et al*

# Supplementary Figures and Tables

#

# Supplementary Table 5A: Clinical Characteristics of Patients with *IKZF1* mutations

| **Characteristics** | **IKZF1^MUT^** | **IKZF1^NEG^** | **OR**  **CI 95%** | ***p-value** |
| --- | --- | --- | --- | --- |
| **Sex** | **N=74** | **N=132** |  |  |
| Male | 43 (58.1%) | 67 (50.8%) | 1.3457  0.7578 to 2.3895 | 0.31 |
| Female | 31 (41.9%) | 65 (49.2%) |  |  |
| **Age at diagnosis** | **N=65** | **N=122** |  |  |
| < 10 | 28 (43.1%) | 87 (71.3%) | 3.2847  1.7521 to 6.1578 | 0.0002 |
| ≥ 10 | 37 (56.9%) | 35 (28.7%) |  |  |
| **WBC at diagnosis** | **N=65** | **N=120** |  |  |
| < 10,000 | 29 (44.6%) | 53 (44.2%) |  |  |
| 10,000 to < 20,000 | 11 (17%) | 25 (20.8%) | 0.8041  0.3467 to 1.8652 | 0.61 |
| 20,000 < 100,000 | 19 (29.2%) | 34 (28.3%) | 1.0213  0.4965 to 2.1007 | 0.95 |
| ≥ 100000 | 6 (9.2%) | 8 (6.7%) | 1.3707  0.4335 to 4.3344 | 0.59 |
| **% of blast at diagnosis in bone marrow** | **N=65** | **N=120** |  |  |
| 20 % to 50% | 3 (4.6%) | 10 (8.3%) | 1.8788  0.4983 to 7.0842 | 0.35 |
| ≥ 50% | 62 (95.4%) | 110 (91.7%) |  |  |
| **Risk classification at diagnosis** | **N=65** | **N=117** |  |  |
| Standard | 17 (26.2%) | 50 (42.7%) | 2.1071  1.0853 to 4.0910 | 0.02 |
| High Risk | 48 (73.8%) | 67 (57.3%) |  |  |

Note: *Fisher’s exact or chi-square test, OR: Odds Ratio, CI: Confidence interval, N: number of patients with available data for each category, WBC: white blood cell count.


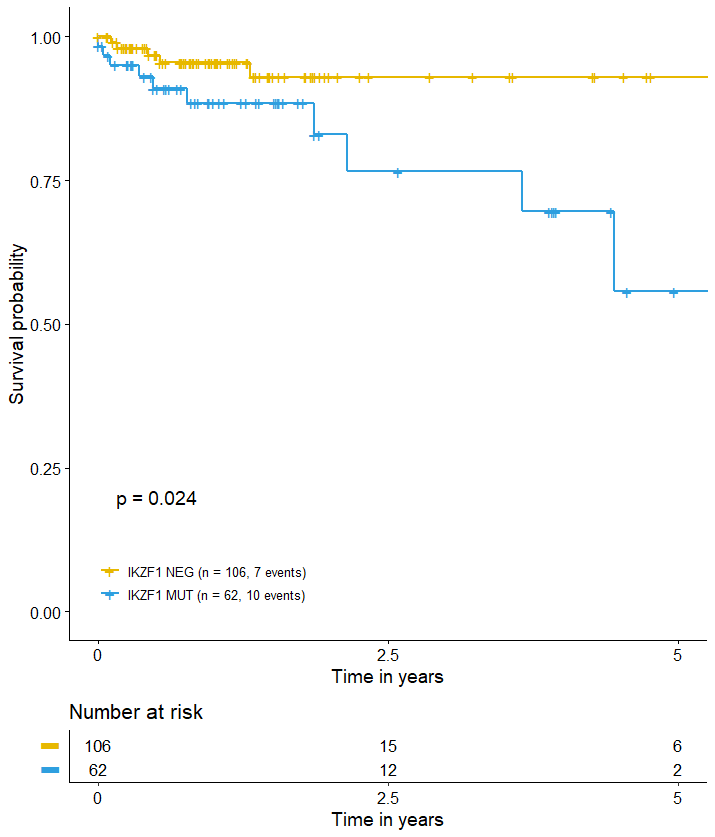


**Figure Supplementary 1A: Overall Survival (OS) Analysis of patients with *IKZF1* mutations:** All patients with *IKZF1MUT* (Blue) were analyzed by Kaplan-Meir overall survival analysis versus those patients *IKZF1NEG* (Yellow).

**Supplementary Table 5B:** Clinical Characteristics of Patients with *CDKN2A/2B* mutations

| **Characteristics** | **CDKN2A/2B^MUT^** | **CDKN2A/2B^NEG^** | **OR**  **CI 95%** | ***p-value** |
| --- | --- | --- | --- | --- |
| **Sex** | **N=79** | **N=127** |  |  |
| Male | 42 (53.2%) | 68 (53.5%) | 0.9849  0.5609 to 1.7293 | 0.95 |
| Female | 37 (46.8%) | 59 (46.5%) |  |  |
| **Age at diagnosis** | **N=70** | **N=117** |  |  |
| < 10 | 37 (52.9%) | 78 (66.7%) | 0.5606  0.3056 to 1.0283 | 0.06 |
| ≥ 10 | 33 (47.1%) | 39 (33.3%) |  |  |
| **WBC at diagnosis** | **N=69** | **N=116** |  |  |
| < 10,000 | 24 (34.8%) | 58 (50%) |  |  |
| 10,000 to < 20,000 | 13 (18.8%) | 23 (19.8%) | 1.3659  0.5956 to 3.1324 | 0.46 |
| 20,000 < 100,000 | 26 (37.7%) | 26 (22.4%) | 2.4167  1.1735 to 4.9766 | 0.01 |
| ≥ 100000 | 6 (8.7%) | 9 (7.8%) | 1.6111  0.5167 to 5.0239 | 0.41 |
| **% of blast at diagnosis in bone marrow** | **N=69** | **N=116** |  |  |
| 20 % to 50% | 1 (1.4%) | 12 (10.3%) | 7.8462  0.9972 to 61.7336 | 0.05 |
| ≥ 50% | 68 (98.6%) | 104 (89.7%) |  |  |
| **Risk classification at diagnosis** | **N=67** | **N=115** |  |  |
| Standard | 20 (29.9%) | 47 (40.9%) | 1.6243  0.8548 to 3.0863 | 0.13 |
| High Risk | 47 (70.1%) | 68 (59.1%) |  |  |

Note: *Fisher’s exact or chi-square test, OR: Odds Ratio, CI: Confidence interval, N: number of patients with available data for each category, WBC: white blood cell count.

**
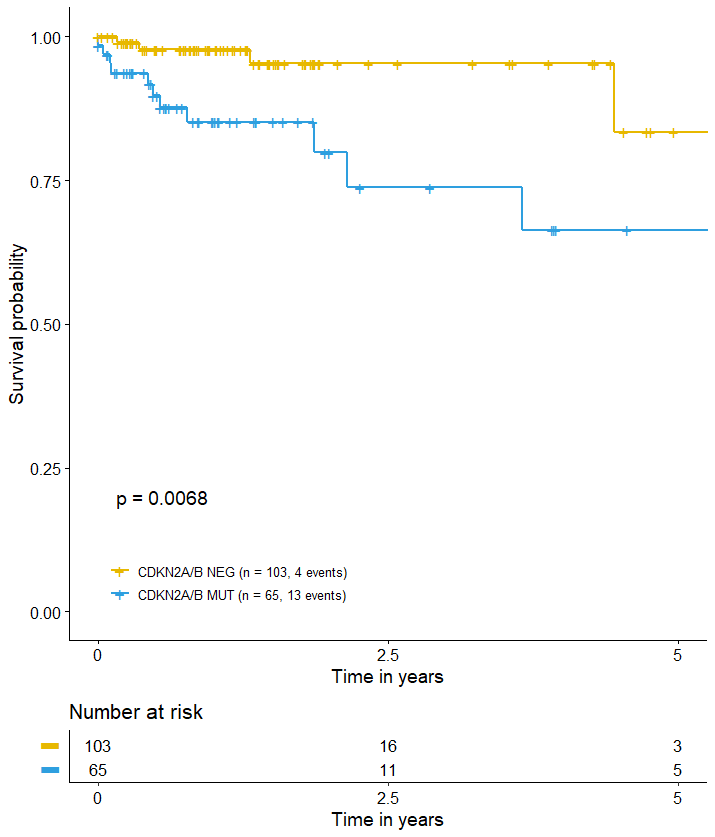
**

**Figure Supplementary 1B: Overall Survival (OS) Analysis of patients with *CDKN2A* mutations:** All patients with *CDKN2A/2B^MUT^* (Blue) were analyzed by Kaplan-Meir overall survival analysis versus those patients *CDKN2A/2B^NEG^* (Yellow).

**
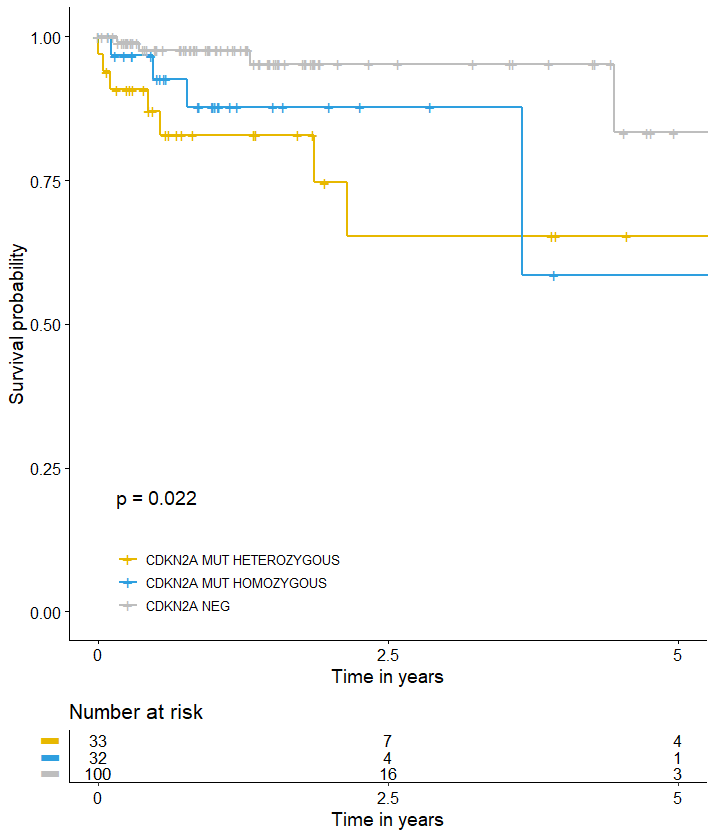
**

**Figure Supplementary 1C:** A) Overall Survival (OS) analyzed by Kaplan-Meir method. Analysis of patients with *CDKN2A* heterozygous deletions (yellow) vs homozygous deletions (blue) and those with *CDKN2A* NEG (grey).

**Supplementary Table 5C:** Clinical Characteristics of Patients with *PAX5* mutations

| **Characteristics** | **PAX5^MUT^** | **PAX5^NEG^** | **OR**  **CI 95%** | ***p-value** |
| --- | --- | --- | --- | --- |
| **Sex** | **N=54** | **N=152** |  |  |
| Male | 30 (55.6%) | 80 (52.6%) | 1.1250  0.6027 to 2.0999 | 0.7 |
| Female | 24 (44.4%) | 72 (47.4%) |  |  |
| **Age at diagnosis** | **N=49** | **N=138** |  |  |
| < 10 | 27 (55.1%) | 88 (63.8%) | 0.6973  0.3599 to 1.3510 | 0.2 |
| ≥ 10 | 22 (44.9%) | 50 (36.2%) |  |  |
| **WBC at diagnosis** | **N=49** | **N=136** |  |  |
| < 10,000 | 22 (44.9%) | 61 (44.8%) |  |  |
| 10,000 to < 20,000 | 8 (16.3%) | 27 (19.9%) | 0.8215  0.3250 to 2.0768 | 0.67 |
| 20,000 < 100,000 | 13 (26.5%) | 39 (28.7%) | 0.9242  0.4175 to 2.0461 | 0.84 |
| ≥ 100000 | 6 (12.3%) | 9 (6.6%) | 1.8485  0.5899 to 5.7927 | 0.29 |
| **% of blast at diagnosis in bone marrow** | **N=49** | **N=136** |  |  |
| 20 % to 50% | 2 (4.1%) | 12 (8.8%) | 2.2742  0.4904 to 10.5464 | 0.29 |
| ≥ 50% | 47 (95.9%) | 124 (91.2%) |  |  |
| **Risk classification at diagnosis** | **N=47** | **N=135** |  |  |
| Standard | 12 (25.5%) | 55 (40.7%) | 2.0052  0.9566 to 4.2032 | 0.06 |
| High Risk | 35 (74.5%) | 80 (59.3%) |  |  |

Note: *Fisher’s exact or chi-square test, OR: Odds Ratio, CI: Confidence interval, N: number of patients with available data for each category, WBC: white blood cell count.

**
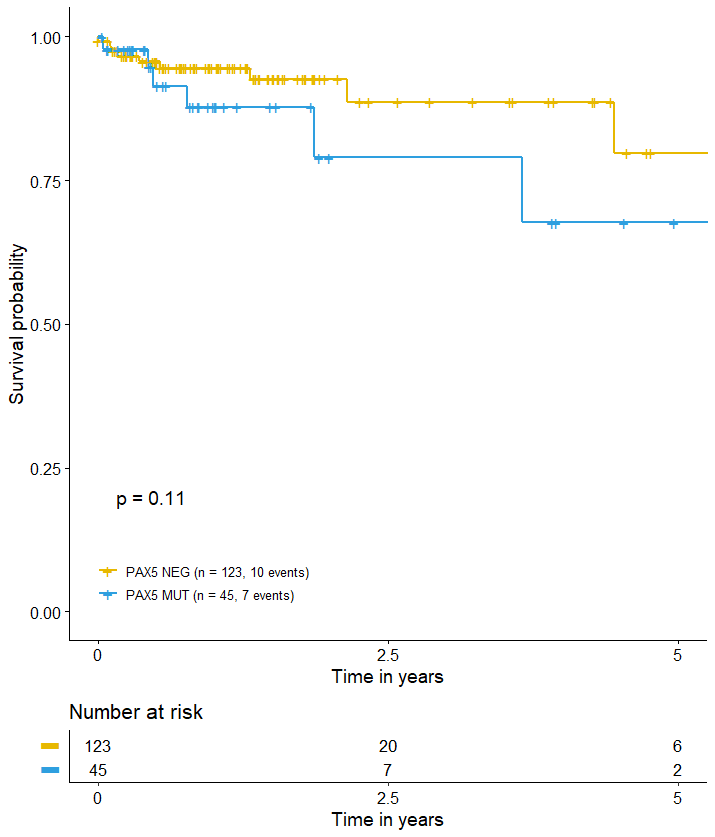
**

**Figure Supplementary 1D: Overall Survival (OS) Analysis of patients with *PAX5* mutations.** All patients with PAX5^MUT^ (Blue) were analyzed by Kaplan-Meir overall survival analysis versus those patients PAX5^NEG^ (Yellow).

**Supplementary Table 5C:** Clinical Characteristics of Patients with *ERG* mutations

| **Characteristics** | **ERG^MUT^** | **ERG^NEG^** | **OR**  **CI 95%** | ***p-value** |
| --- | --- | --- | --- | --- |
| **Sex** | **N=18** | **N=188** |  |  |
| Male | 11 (61.1%) | 99 (52.7%) | 1.4127  0.5250 to 3.8017 | 0.49 |
| Female | 7 (38.9%) | 89 (47.3%) |  |  |
| **Age at diagnosis** | **N=18** | **N=169** |  |  |
| < 10 | 11 (61.1%) | 104 (61.5%) | 1.0182  0.3757 to 2.7595 | 0.97 |
| ≥ 10 | 7 (38.9%) | 65 (38.5%) |  |  |
| **WBC at diagnosis** | **N=18** | **N=167** |  |  |
| < 10,000 | 9 (50%) | 74 (44.3%) |  |  |
| 10,000 to < 20,000 | 3 (16.7%) | 32 (19.2%) | 0.7708  0.1957 to 3.0362 | 0.70 |
| 20,000 < 100,000 | 4 (22.2%) | 48 (28.7%) | 0.6852  0.1998 to 2.3502 | 0.54 |
| ≥ 100000 | 2 (11.1%) | 13 (7.8%) | 1.2650  0.2450 to 6.5319 | 0.77 |
| **% of blast at diagnosis in bone marrow** | **N=18** | **N=167** |  |  |
| 20 % to 50% | 2 (11.1%) | 11 (7%) | 0.5641  0.1148 to 2.7720 | 0.48 |
| ≥ 50% | 16 (88.9%) | 156 (93%) |  |  |
| **Risk classification at diagnosis** | **N=18** | **N=164** |  |  |
| Standard | 8 (44.4%) | 59 (36%) | 0.7024  0.2629 to 1.8769 | 0.48 |
| High Risk | 10 (55.6%) | 105 (64%) |  |  |

Note: *Fisher’s exact or chi-square test, OR: Odds Ratio, CI: Confidence interval, N: number of patients with available data for each category, WBC: white blood cell count.

**
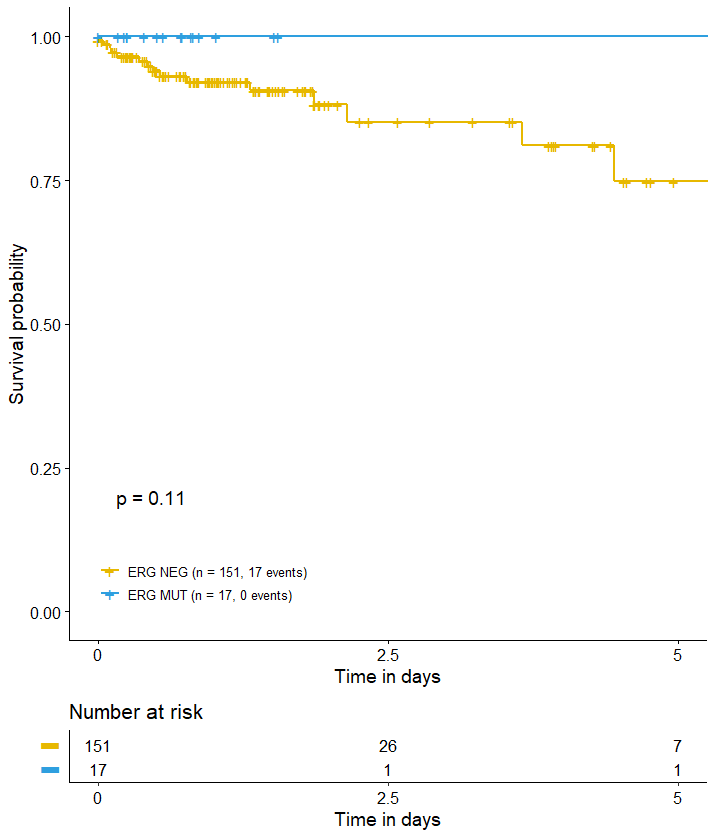
**

**Figure Supplementary 1E: Overall Survival (OS) Analysis of patients with *ERG* mutations:** All patients with ERG^MUT^ (Blue) were analyzed by Kaplan-Meir overall survival analysis versus those patients ERG^NEG^ (Yellow).

**A**

**B**


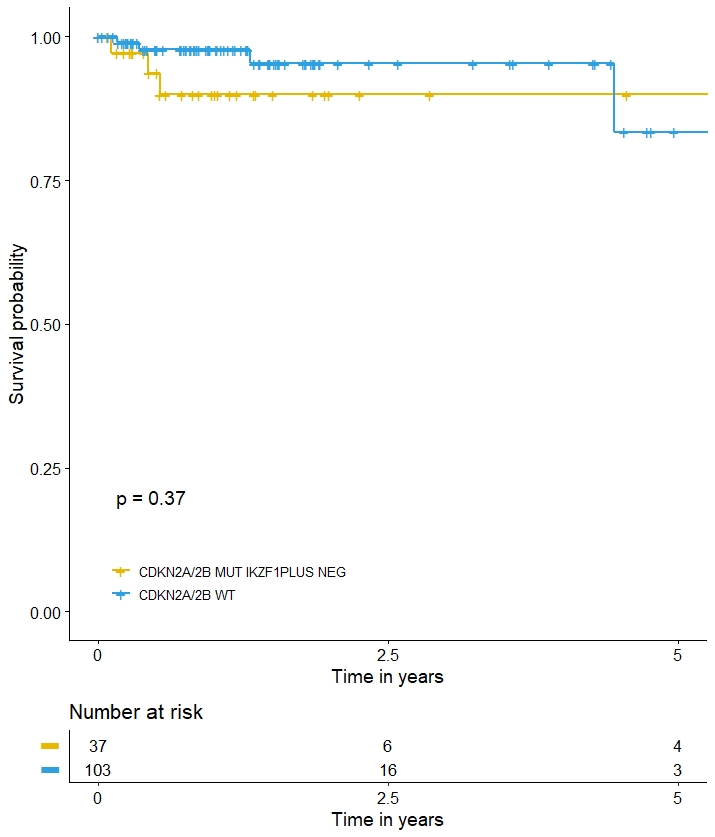

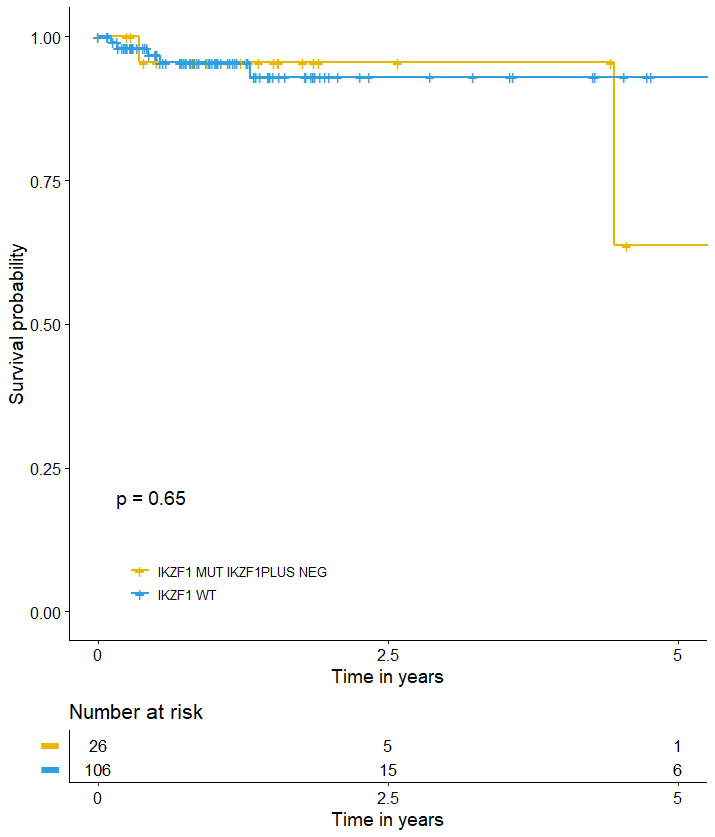


**Figure Supplementary 2: A) Overall Survival (OS) Analysis of patients with *IKZF1* and B) *CDKN2A/2B* mutations without the *IKZF1^plus^* profile A)** All patients with *CDKN2A/2B^MUT^* (Blue) were analyzed by Kaplan-Meir overall survival analysis versus those patients *CDKN2A/2B^NEG^* (Yellow). **B)** All patients with *IKZF1^MU^*^T^ (Blue) were analyzed by Kaplan-Meir overall survival analysis versus those patients *IKZF1^NEG^* (Yellow).
